# Gastric and cardiac inflammatory myofibroblastic tumor: an extremely rare case

**DOI:** 10.1186/s13019-024-02481-9

**Published:** 2024-01-29

**Authors:** Yueqi Huang, Mingqi Zhang, Qingchun Li, Qiulin Huang

**Affiliations:** 1 Department of Ultrasound Medicine, The First Affiliated Hospital, Hengyang Medical School, University of South China, Hengyang, 421001 Hunan People’s Republic of China; 2 Department of General Surgery, The First Affiliated Hospital, Hengyang Medical School, University of South China, Hengyang, 421001 Hunan People’s Republic of China; 3 Department of Image, The First Affiliated Hospital, Hengyang Medical School, University of South China, Hengyang, 421001 Hunan People’s Republic of China

**Keywords:** Gastric, Cardiac, Inflammatory myofibroblastic tumor (IMT), Prognosis, Activin receptor-like kinase (ALK)

## Abstract

**Background:**

Inflammatory myofibroblastic tumor (IMT) is a unique, rarely metastatic tumor composed of myofibroblasts and fibrous spindle cells with inflammatory cell infiltration that can affect any organ in the human body. By reviewing the relevant literature on PubMed, we found that this is the first case report of IMT with both gastric and cardiac involvement.

**Case presentation:**

A 57-year-old male patient was admitted to the hospital with complaints of malaise, poor appetite, and epigastric pain with black stools. We found a mass in the patient’s stomach and left atrium by contrast-enhanced computed tomography, 18 F-fluorodeoxyglucose positron emission tomography/computed tomography, and other tests. The patient underwent laparoscopic Billroth II subtotal gastrectomy and Braun’s gastrointestinal reconstruction under general anesthesia. On the 46th day following stomach surgery, the cardiac tumor was removed under general anesthesia. The patient has treated with doxorubicin 70 mg of D1 chemotherapy two months after cardiac surgery. Postoperative pathological immunohistochemistry of the mass confirmed the diagnosis of an IMT. His review three months after the cardiac surgery suggested the progression of the left atrial mass, but he declined further treatment and finally died one month after the review.

**Conclusions:**

As a unique class of tumors that rarely metastasize, IMTs have an unknown etiology and pathogenesis, and distant metastasis is primarily observed in patients with negative activin receptor-like kinase (ALK) expression. The preferred treatment for IMT is complete surgical resection, and the effectiveness of adjuvant therapy for patients with distant metastases is still being determined. The clinical presentation of IMT lacks specificity and is often related to the location of tumor growth, which poses a diagnostic challenge. Pathological immunohistochemistry is the only way to confirm the diagnosis at present. Our case report reminds clinicians that a category of ALK-negative IMT with a tendency toward distant metastasis should not be ignored.

## Background

The latest World Health Organization (WHO) definition of inflammatory myofibroblastic tumor (IMT) in 2020 indicates that it is a distinctive, rarely metastatic neoplasm consisting of myofibroblasts and fibroblastic spindle cells with inflammatory cell infiltration, first described by Birch-Hirschfield in 1905 [1, 2]. IMT can affect any body organ and is more common in patients under 16 than adults [2, 3]. Due to the low incidence, especially in adults, and lack of specificity in clinical and radiological presentations, IMTs are frequently misdiagnosed [4]. As a less metastatic tumor, IMT has a good prognosis for patients who can be surgically resected, with 5- and 10-year survival rates of 91% and 77%, respectively [5]. By reviewing the relevant literature on PubMed, we found that this is the first case report of IMT with both gastric and cardiac involvement.

## Case presentation

**Fig. 1:**
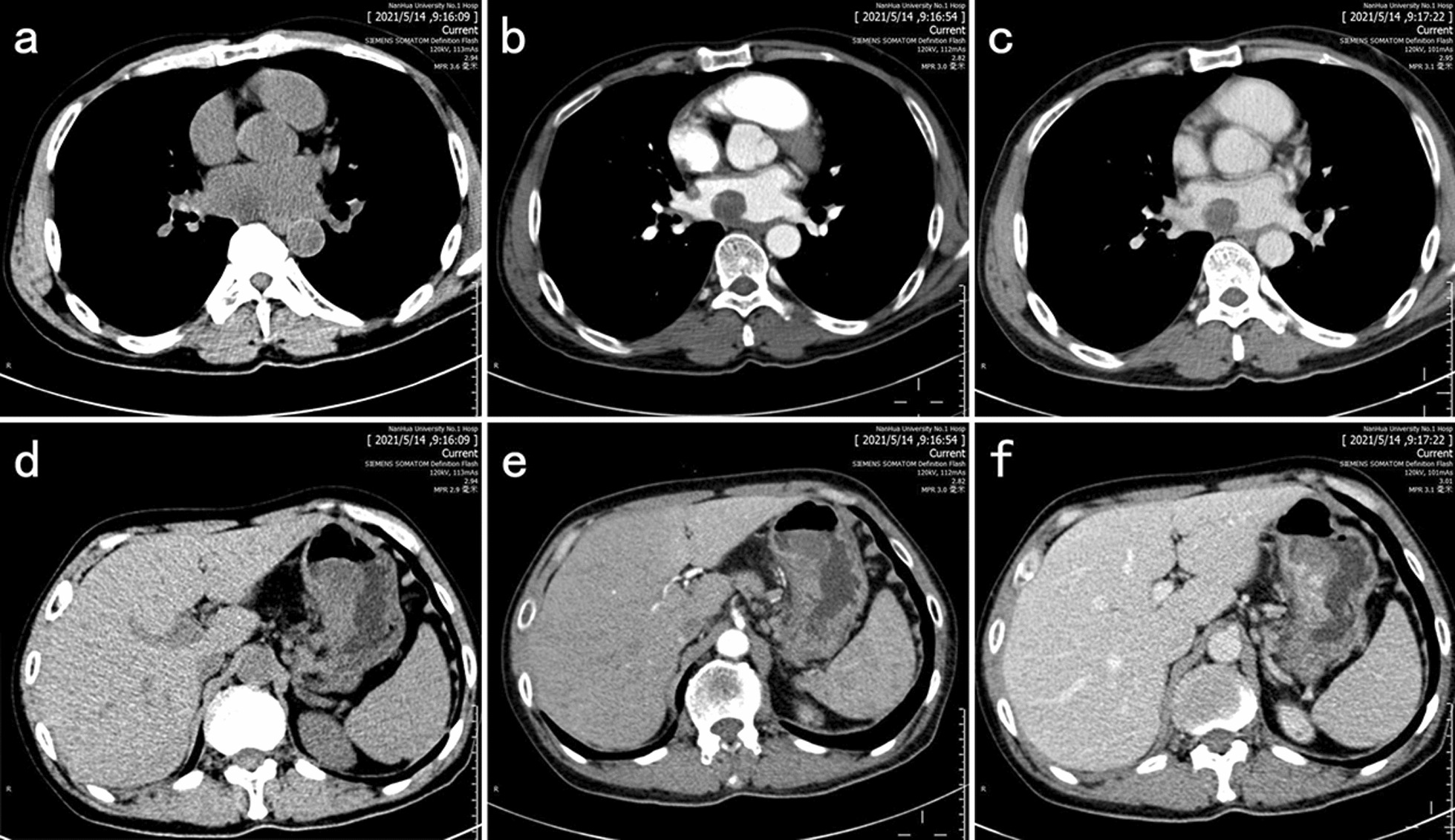
**a** On chest scan, a spherical/flat mound-shaped mass growing prominently into the left atrium with a broad base attached to the posterior wall of the left atrium is seen; **b + c** on enhancement scans, the mass is moderately enhanced, and trophoblastic vessels are seen within it. **d** On abdominal scan, a mass-like thickening with a cauliflower-like shape growing into the gastric lumen is seen in the gastric wall on the side of the gastric lesser curvature; **e + f** On enhancement scans, the mass moderately but progressively strengthens with the interrupted inward lifting of the mucosal line and thickening of the submucosal edema. Multiple lymph nodes are seen next to the right gastric vein in the lesser curvature of the stomach

**Fig. 2:**
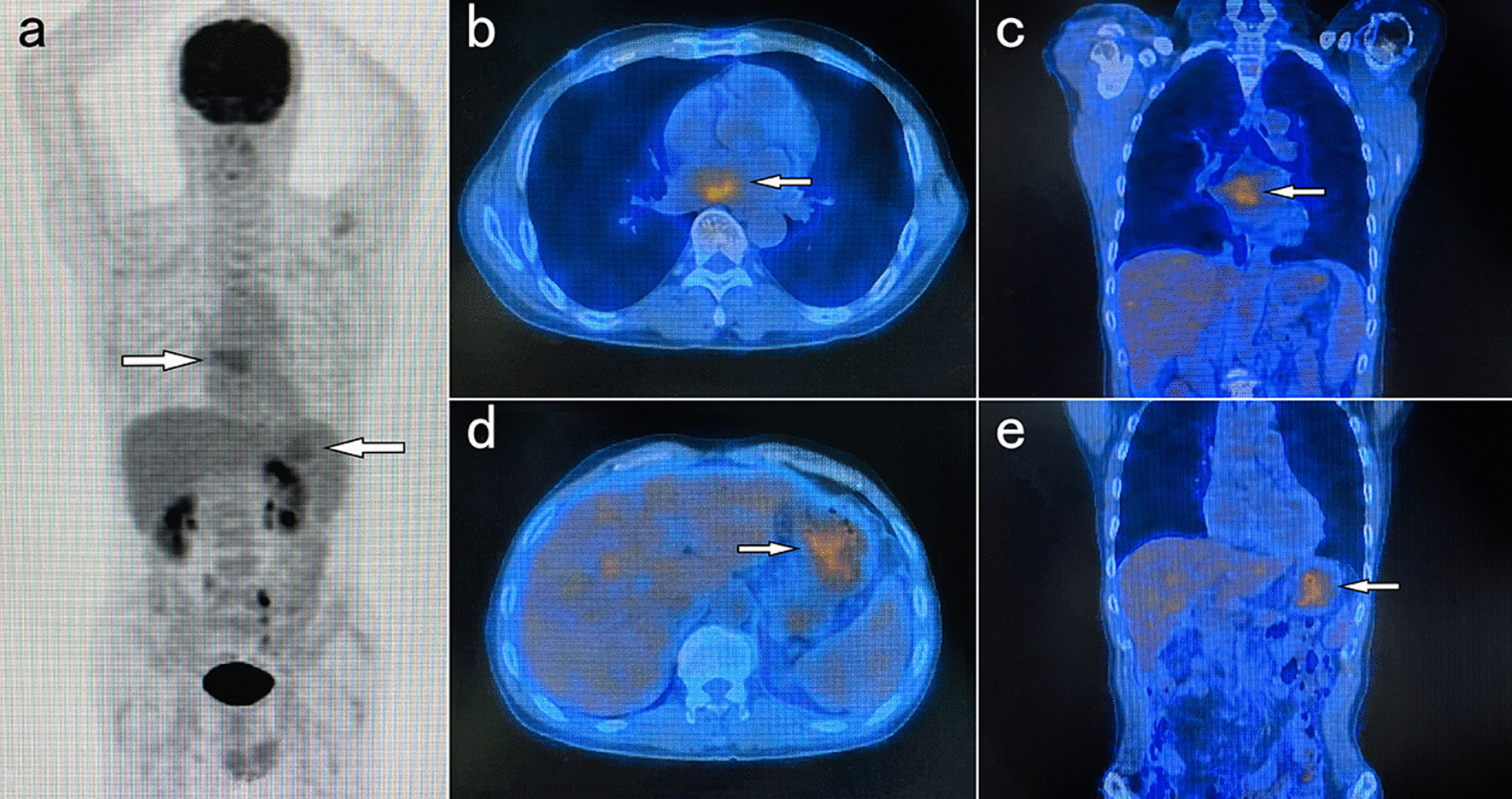
**a** Maximum-intensity projection images showing increased uptake of radiotracer in the gastric and cardiac regions (arrow); **b + c** Axial and coronal fusion images showing increased cardiac radiotracer uptake (arrow), placing the region of interest (ROI) in an elevated cardiometabolic shadow with a maximum standardized update value (SUVmax) of approximately 3.1; **d + e** Axial and coronal fusion images showing increased gastric radiotracer uptake (arrow), with a SUVmax of 3.27 when the ROI was placed in the metabolically elevated shadow of the gastric mucosa adjacent to the gastric mass

**Fig. 3:**
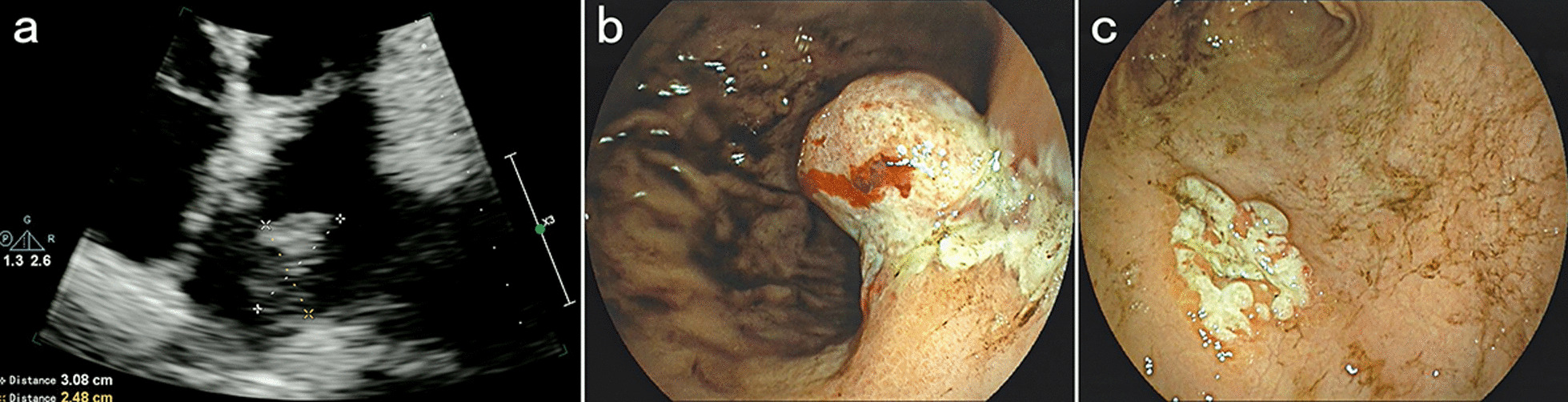
**a** The left atrium is slightly enlarged, and a hypoechoic mass of approximately 31 × 25 mm is observed at the top of the left atrium, which does not oscillate with the cardiac cycle. **b** There is a polypoid mass with an erosive surface of approximately 3 × 4 cm in size on the lesser curvature of the gastric body, and ulcers are distributed around its root with white moss on its surface; **c** The mucosa at the gastric sinus is red and white with scattered small flakes of erosion, and the thickened elevation of the mucosa at its anterior wall near the greater curvature of the stomach is approximately 2.0 × 1.5 cm in size, with a white, uneven surface

A 57-year-old male patient was admitted to the hospital with complaints of malaise, poor appetite, and epigastric pain with black stools. Physical examination of the patient revealed that he was pale, abdominal palpation revealed epigastric tenderness and cardiac murmurs could be heard on cardiac auscultation. He had a previous history of *H. Pylori* infection and gastric ulcer for more than a year and was treated with standard anti-*H. Pylori* treatment for a fortnight. Laboratory tests: RBC 2.57 × 10^12^/L, hemoglobin 50 g/L, C-reactive protein 41.5 mg/L, mean corpuscular volume (MCV) 73.7 fL, mean corpuscular hemoglobin (MCH) 22.3 pg, mean corpuscular hemoglobin concentration (MCHC) 303 g/L, albumin 28.40 g/L, transferrin 15.5 µmol/L, and iron 6.33 µmol/L. Contrast-enhanced computed tomography revealed a spherical/flat mass in the left atrium growing convexly into the left atrium (Fig. 1a–c), together with a cauliflower-like mass–like thickening of the gastric wall of the lesser curvature of the stomach growing into the gastric cavity (Fig. 1d–f). 18 F-Fluorodeoxyglucose (18 F-FDG) positron emission tomography/computed tomography (PET/CT) showed (1) an enlarged left atrium with a faint mass of increased metabolism, the nature of which was to be determined (Fig. 2a–c); and (2) a mass at the junction of the gastric antrum and body growing toward the lumen of the stomach, with increased metabolism in the adjacent gastric mucosa, which we considered a highly differentiated gastric cancer or benign lesion (Fig. 2a, d, and e). Cardiac ultrasound showed a hypoechoic mass of undetermined nature at the top of the left atrium (Fig. 3a). Electronic gastroscopy showed a polypoid mass with a surface erosion of approximately 3 × 4 cm in size at the lesser curvature of the gastric body (Fig. 3b); the mucosa was seen to be red and white at the gastric sinus, and its mucosal elevation near the greater curvature of the stomach was approximately 2.0 × 1.5 cm in size (Fig. 3c). Gastroscopic biopsies of the gastric body’s polyps and the gastric sinus’s mucosa were taken five times each, revealing the presence of inflammatory necrotic material and chronic superficial inflammation of the gastric mucosa. A multidisciplinary team consultation was carried out on this patient. Based on the PET/CT results, it was inferred that the cardiac mass, whose nature was currently unclear, was considered to be homologous to the gastric mass, while distant metastases to other vital organs of the body, such as the lungs, liver, and brain, could be excluded. Temporary anticoagulation with 4000 U low-molecular-weight heparin calcium subcutaneously q12h was given to prevent thrombosis. As the patient had gastrointestinal bleeding symptoms and anemia was considered grade 4 according to the World Health Organization classification, we finally decided to deal with the gastric mass first, then the cardiac mass after the patient’s anemic state had recovered after the gastric surgery.

**Fig. 4:**
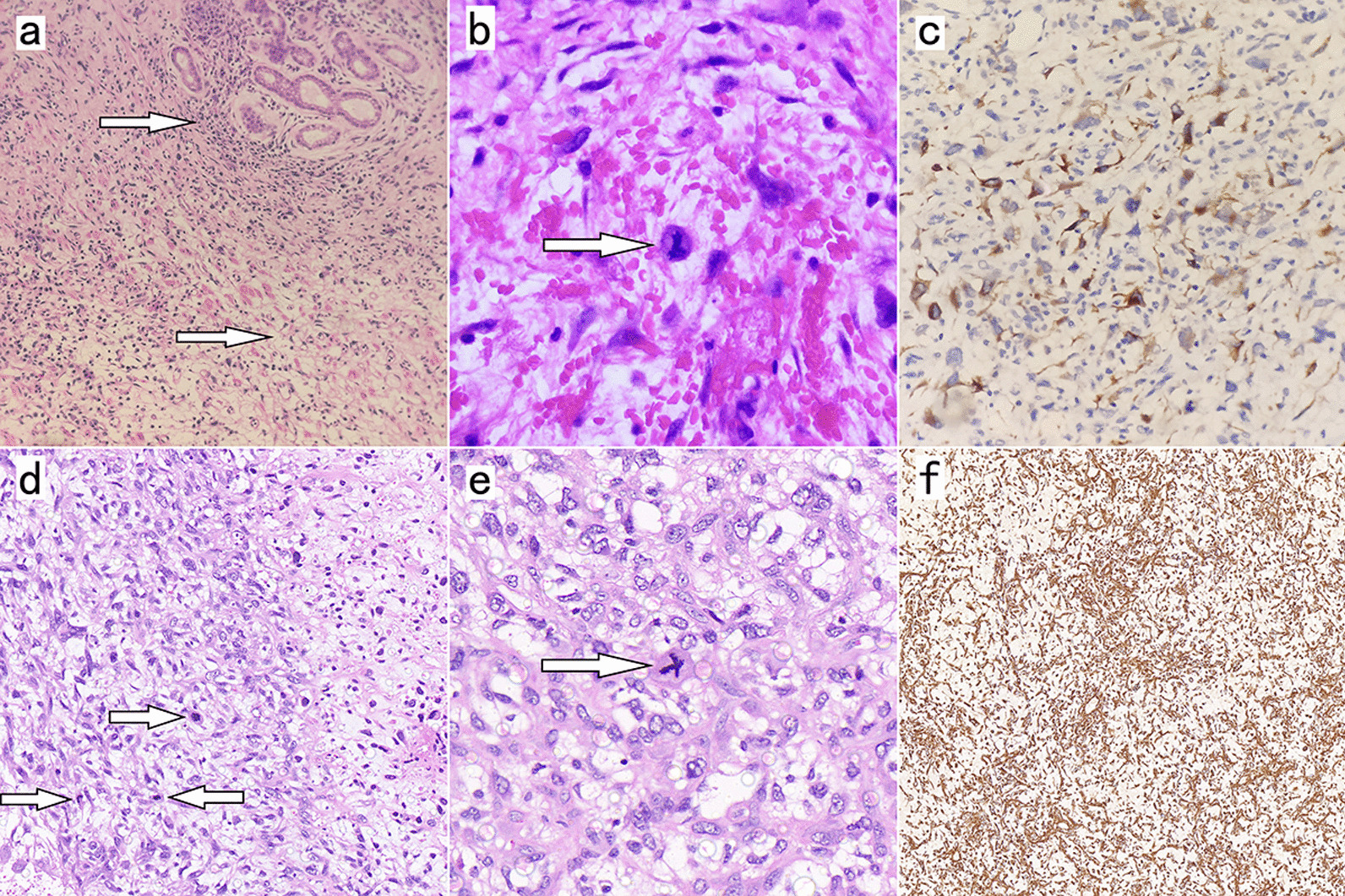
**a** Resected gastric mass showing inflammatory cell infiltration around normal gastric mucosal tissue (upper arrow) and mucoid degeneration (lower arrow) visible in the tumor interstitium (H&E×100); **b** Tumor cells of the stomach (arrow) have cellular atypia and scattered mitotic figures (H&E×400); **c** Desmin exhibits diffuse positive expression in resected gastric tumor specimens. **d** Left atrium resected mass shows large, round, polygonal, and spindle-shaped tumor cells (arrows) that are higher in density (H&E×200) than masses of gastric origin; **e** Tumor cells of the heart (arrow) have cellular atypia and scattered mitotic figures (H&E×400); **f** Vimentin exhibited diffuse positive expression in resected cardiac tumor specimens

The patient underwent laparoscopic Billroth II subtotal gastrectomy and Braun’s gastrointestinal reconstruction under general anesthesia. Microscopically (Fig. 4a and b), scattered spindle-shaped tumor cells with cellular anisotropy and abundant, red-stained cytoplasm were observed, with a sporadic mitotic figures (1–2 per 10 HPF). The tissue was infiltrated with inflammatory cells, mainly lymphocytes, with a mucus-rich and hemorrhagic interstitium. Immunohistochemistry showed that the tumor cells were positive for Desmin (Fig. 4c) and β-catenin but negative for Actin, S-100, EMA, ALK, CD68, Dog-1, and CD117. The Ki-67 proliferative activity index of tumor cells was 5%. The mouse double minute 2 (MDM2) fluorescence in situ hybridization (FISH) assay was positive for tumor cells, that is, MDM2 gene amplification. Taking these results together, we considered the patient’s stomach tumor IMT. The patient recovered well after surgery.

**Fig. 5:**
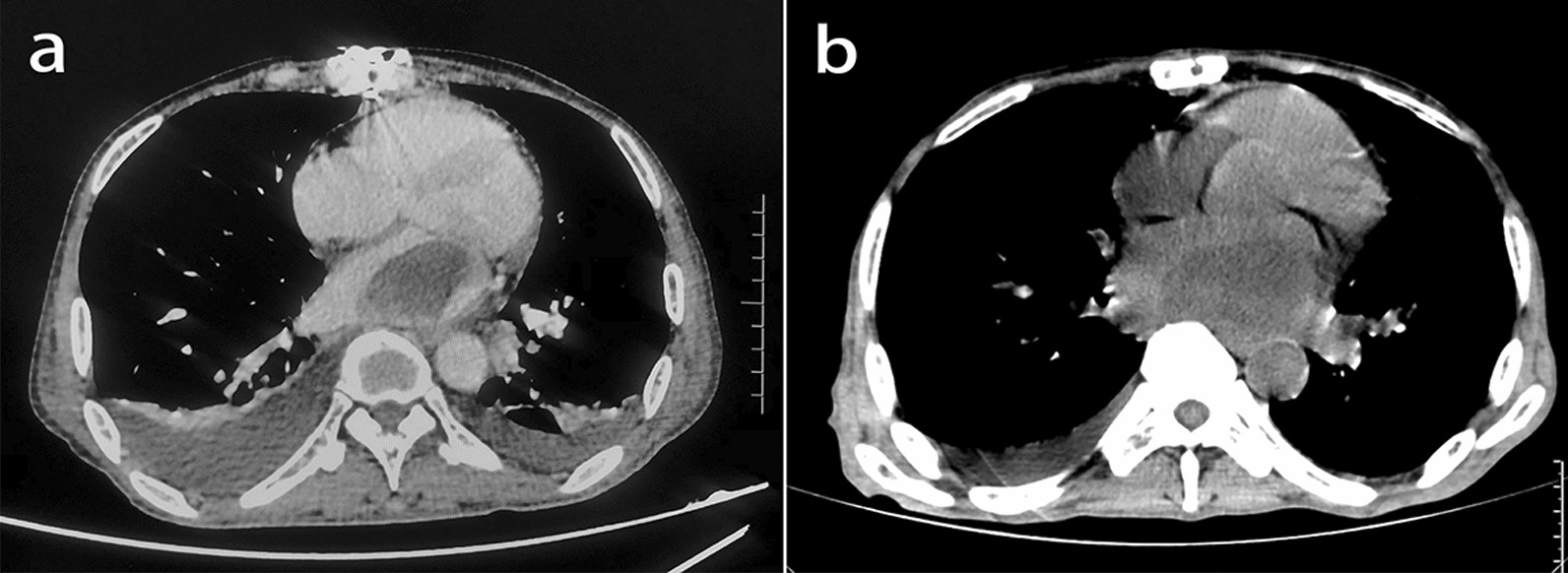
**a** The left atrium has an oval-shaped tumor measuring 4.8 × 3.2 cm with a broad base and proximity to the posterior wall. Internal fixation and associated artifacts are observed at the sternum, and there is a small amount of bilateral pleural effusion; **b** The bilateral pleural effusion has been decreased, the internal fixation at the sternum has been removed, and the oval hypodense mass in the left atrium is larger than before (5.5 × 3.8 cm)

To avoid more serious complications, such as thrombosis and inflow obstruction, we performed cardiac tumor removal under general anesthesia on the 46th day after gastric surgery due to the continued growth of the cardiac mass. During the surgery, we found that the tumor was broad basally and could not be eradicated, so we had to excise as much of the mass as possible along the tumor’s root and cauterize the cut edge with an electrotome. Postoperative review of contrast-enhanced CT of the chest (Fig. 5a) showed an oval tumor (4.8 × 3.2 cm) in the left atrium with a broad base in close contact with the posterior wall. Postoperative pathology (Fig. 4d and e) showed large, round, polygonal, and spindle-shaped tumor cells with thin and coarse nuclear chromatin, cellular pleomorphism, and a higher density of mitotic images (30 per 10 HPF) than that observed in stomach-derived masses. Immunohistochemistry showed that the tumor cells were positive for Vimentin (Fig. 4f), MDM, SMA, Bcl-2, Ki67, and CD99 and negative for TLE1, S-100, STAT6, CD34, ALK, CK-Pan, CD68, and ERG. The Ki-67 proliferative activity index of tumor cells was 10%. Therefore, the patient’s heart mass was also diagnosed as IMT. The patient was discharged from the hospital with an average postoperative recovery.

The patient has treated with doxorubicin 70 mg of D1 chemotherapy two months after cardiac surgery. He had a repeat chest CT (Fig. 5b) 3 months after cardiac surgery, which showed an oval-shaped hypodense tumor in his left atrium that was larger than before (5.5 × 3.8 cm). Finally, he refused further treatment and died one month after the review.

## Discussion and conclusions

As a unique class of neoplasms that seldom metastasize, IMT has an unclear etiology and pathogenesis. The etiology of IMT is generally related to surgery, trauma, inflammation, abnormal repair, or unique infections such as human herpesvirus-8 [2]. Cytogenetic translocation of the ALK (anaplastic lymphoma kinase) locus on 2p23 is observed in 50-60% of cases of IMT, and rearrangements of other genes are also detected in cases of IMT, such as ROS1, platelet-derived growth factor receptor beta (PDGFRB), RET, etc. [6, 7]. . However, no association was found between specific genetic ectopics and the patient’s age, sex, or anatomical localization of the tumor [7]. The patient’s preoperative blood C-reactive protein was higher than normal, so we considered that his illness might be related to the inflammatory response caused by gastric tumor rupture and bleeding.

Complete surgical resection is the optimal course of action for IMT. Given the benign nature of IMT, partial resection is also advised even when the tumor invades vital tissues in the chest or abdomen and cannot be removed entirely [1, 8, 9]. However, we cannot ignore a rare class of malignant IMT characterized by significant clinical deterioration and aggressive tumor behavior with recurrence and/or metastasis [1]. In addition to surgery, these patients need to receive some adjuvant treatments, such as hormones, NSAIDs, chemotherapy, and radiotherapy [1, 5, 8, 10]. The recent CREATE study on IMT (EORTC 90,101, NCT01524926) confirmed through long-term follow-up efficacy analysis that crizotinib, a small-molecule tyrosine kinase inhibitor targeting ALK, MET, ROS1, and RON, is highly effective and provides durable benefit in patients with locally advanced or metastatic ALK-positive IMT who do not qualify for curative surgery [11, 12]. Distant metastases were primarily observed in patients with negative ALK expression. None of the above adjuvant therapies were satisfactory, suggesting that ALK expression is associated with a better clinical prognosis [5].

The patient, in this case, received doxorubicin monotherapy rather than doxorubicin combined with ifosfamide, per the CREATE study (EORTC 62,012), for the following reasons [13, 14]: (1) Intensive combination chemotherapy with doxorubicin plus ifosfamide did not improve overall survival compared with doxorubicin alone. (2) The toxicity of combination therapy is significantly higher than that of monotherapy, and the patient had undergone two surgeries with a poor underlying physical condition. (3) Ifosfamide is more favored for synovial sarcoma and less for leiomyosarcoma. This ALK-negative patient’s tumor continued progressing despite postoperative chemotherapy after two separate surgeries (one radical and one palliative) to completely resect the intragastric IMT and partially remove the patient’s endocardial polyp-like IMT mass, suggesting that complete surgical resection may be the only effective approach for ALK-negative patients.

IMT can affect any organ or tissue in the body, such as the heart, mesentery, bones, uterus, and central nervous system, but the most common is the lung [2, 3]. The lack of specificity in the clinical presentation of IMT, which is often associated with the location of neoplasia growth, poses a diagnostic challenge. Immunohistochemistry is currently the most effective way to diagnose IMT, which is usually positive for vimentin and SMA and negative for CD34, CD117, and S100 [1, 2, 15]. In our experience, for patients who are eligible for pathologic biopsy, taking deeper tissue from the mass and doing immunohistochemistry can help to confirm the diagnosis of IMT preoperatively, while PET-CT can also play a supporting role in the diagnostic process. The nuclear overexpression of MDM2 has been reported to be more frequently detected in IMTs with cellular atypia than in those without [16, 17]. We expect that new molecular markers specific to IMT can be discovered in the future, providing new ways to diagnose IMT more easily and quickly.

## Data Availability

This published article and its supplementary information files include all data generated or analyzed during this study.

## Abbreviations

IMT: Inflammatory myofibroblastic tumor
CT: Computed tomography
18F-FDG PET/CT: 18 F-fluorodeoxyglucose positron emission tomography/computed tomography
HPF: High-power field
ALK: Activin receptor-like kinase
MDM2: Mouse double minute 2
NSAIDs: Nonsteroidal anti-inflammatory drugs
